# Impact of fibrin glue versus suture closure on double-headed pterygia in Asian eyes – a 7-year study in a tertiary institution

**DOI:** 10.1080/07853890.2021.1901304

**Published:** 2021-03-18

**Authors:** Wei Wei Dayna Yong, Liang Shen, Ray Manotosh, Wee Tien Anna Marie Tan, Hui Chen Charmaine Chai

**Affiliations:** a Department of Ophthalmology, National University Hospital, Singapore, Singapore; b Yong Loo Lin School of Medicine, National University of Singapore, Singapore, Singapore

**Keywords:** Double-headed pterygia, conjunctival autograft, fibrin glue, sutures

## Abstract

**Background:**

To compare the recurrence rate and outcomes of double-headed pterygia using fibrin glue versus suture closure of conjunctival autograft.

**Methods:**

All patients with double-headed pterygia who underwent pterygia excision with conjunctival autograft from January 2012 to January 2019 in the National University Hospital of Singapore were included. Patients were divided into 2 groups depending on whether fibrin glue or sutures were used to secure the conjunctival autograft in place. All patients had a minimum of 6 months follow-up.

**Results:**

A total (26 patients) of 22 eyes had fibrin glue, while eight eyes underwent suture closure of their conjunctival autograft. Fibrin glue group had 4.5% recurrence rate, while suture group had 37.5% recurrence rate (*p* = .021). There is statistically significant improvement for overall visual acuity (*p* = .009) and cylinder (*p* = .002). There is also statistically significant improvement for visual acuity in the glue group (*p* = .026), but not in the suture group. Fibrin glue group had a shorter operation duration time compared to suture group (*p* < .001).

There were no cases of graft dislocation, contraction or limbal stem cell deficiency.

**Conclusions:**

Low recurrence rates and good postoperative visual outcomes can be achieved with the split conjunctival autograft technique. Our study suggests that fibrin glue has an additional benefit over the use of sutures in the management of these complex cases.

## Introduction

Pterygia are fibrovascular overgrowth of the Tenon's capsule and bulbar conjunctiva onto the cornea, which may cause ocular surface irritation, tear film-related problems, astigmatism or reduced cosmesis [1,2]. Ultraviolet light has been found to play a key role in its pathogenesis [3]. Double-headed pterygia present as nasal and temporal pterygia in the same eye. These are rare and have a reported incidence of less than 2.5% [4]. The overall worldwide incidence of pterygium is 10.2%, based on a meta-analytic study by Liu et al. [5]. These pterygia tend to cause higher astigmatism and more visual symptoms. They are harder to treat surgically since a larger graft has to be harvested to cover the large exposed scleral after excision. With a large graft, there is potentially a higher risk of inflammation, recurrence and limbal stem cell deficiency.

Various surgical techniques have been described in recent years, be it simultaneous double-headed pterygiectomy or a two-stage surgery [6–8]. The best method is yet unknown. Our technique is similar to that described by Duman et al. [9] using the vertically-split conjunctival autograft technique, who reported good outcomes with no recurrences. Multiple studies have reported good outcomes with the use of conjunctival autografts in the management of pterygium [10,11].

A meta-analysis [12] and a Cochrane review [13] looking at the use of fibrin glue versus suture in conjunctival autografting in the treatment of pterygium both found that glue was superior in reducing the recurrence rate, operating time and improving cosmesis. However, fibrin glue was reported to be associated with increase in complications such as graft dehiscence, retraction and granuloma. These studies excluded double-headed pterygia in their analysis. With the increase in inflammation and recurrence rate expected upon removal of double-headed pterygia, we hypothesize that the use of fibrin glue in double-headed pterygia will lead to better outcome and lower recurrence compared with sutures.

To our knowledge, there are limited reports, with few numbers, on surgical outcomes in the management of double-headed pterygia, with most being case studies. We are also unaware of any other study that has compared the outcomes and recurrence using fibrin glue versus suture in the surgical management of double-headed pterygia. Our study aims to fill this gap by reporting our experience over many years using these techniques.

## Methods

All patients with double-headed pterygiawho underwent pterygia excision with conjunctival autograft from January 2012 to January 2019 in the National University Hospital of Singapore were identified retrospectively from operating theatre records. These patients were divided into two groups depending on whether fibrin glue or sutures were used to secure the conjunctival autograft in place. Patients included had to have a follow-up of at least 6 months for those that had no recurrence of pterygia.

Preoperative data collected included whether it was a primary or recurrent pterygium, surgical indication, preoperative visual acuity and astigmatism. Visual acuity was measured using Snellen chart and converted to Logarithm of the Minimum Angle of Resolution (LogMAR) visual acuity. The severity of the pterygium was graded according to the extent of corneal involvement [14]. The surgical techniques were reviewed to ensure that a similar surgical approach was performed on all included patients. Intraoperatively, the size of the conjunctival graft harvested was recorded in terms of the graft area harvested. Patients were reviewed at various intervals and presence of recurrence was recorded.

The study adhered to the tenets of the Declaration of Helsinki and received approval from the Institutional Review Boards. No consent was required to be taken from the patients.

### Statistical analysis

All variables were analysed descriptive first. Numerical variables were summarized by mean with SD, or median with range, whichever applicable; while categorical variables were summarized by frequency tables. Kaplan–Meire method with log-rank test was used to compare the time to recurrence between glue group and suture group. K–M estimate was used for recurrence rate at XX days. Visual acuity was expressed as LogMAR for statistical analysis. Mann–Whiney U-test was used to compare the postoperative visual outcomes between glue and suture group; while Wilcoxon Signed Rank test was used to compare the visual outcomes between the postoperative measurement and the preoperative measurement. A *p*-value <.05 was considered statistically significant. Statistical analysis was performed using SPSS software version 24.0 for Windows (SPSS, Inc., Chicago, IL).

### Surgical technique

Surgery was performed under peribulbar block. A corneal traction suture was placed superiorly to allow adequate conjunctival manipulation and exposure. The nasal and temporal pterygia were excised close to the limbus. The underlying tenon’s was removed, exposing down to bare scleral. Haemostasis was achieved with gentle cautery of the bleeding vessels. A pterygium blade was used to clear the fibrovascular tissue over the cornea. The bare scleral defects were measured with a calliper and the total graft size calculated and marked with surgical ink over the superior bulbar conjunctival. A thin conjunctival graft was carefully dissected free from the underlying tenon’s and vertically split to the appropriate size as previously measured. Grafts were carefully transferred over the temporal and the nasal defects and secured in place either with either fibrin glue or 8/0 vicryl sutures. Limbus to limbus orientation was maintained. A bandage contact lens was placed. No adjunctive agents were used in all our cases.

Postoperatively, the patients were treated with combination drops of topical steroid and antibiotics. This medication was started at 3 hourly intervals at postoperative day 1 and continued for about a week. This was then slowly tapered and stopped over 1 to 2 months. Patients were followed up at postoperative day 1, week 1 and month 1 and variable number of months after. Postoperative best corrected visual acuity was taken at 6 months postoperatively. Postoperative complications and recurrences were recorded. Recurrence was defined as new fibrovascular growth over the limbus. No suture removal was performed unless the sutures were loose and causing secondary irritation.

## Results

A total of 39 eyes (36 patients) underwent surgery for double-headed pterygia during the study period of January 2012 to January 2019. Of these, three eyes are excluded as there was lost to follow up after first day postoperatively, four eyes are excluded as their surgeries were not done by a cornea surgeon and two other eyes are excluded as they underwent primary conjunctival closure of the smaller pterygia. Of the remaining eyes (26 patients), 22 eyes received fibrin glue, while eight eyes had their conjunctival autograft secured with sutures. Surgeries of all eyes which received fibrin glue were performed or supervised by either of two cornea consultants (Anna Tan, Ray Manotosh). The baseline characteristics of these patients are included in Table 1. Of note, the patients in the fibrin glue group had more advanced pterygia with the need for larger autografts.

**Table 1.** Baseline characteristics and clinical results.

Variable	Total	Fibrin Glue Group	Suture Group	*p* Value
Age (years)				
Mean (SD)	65.68 (12.486)	62.88 (13.564)	71.63 (7.386)	.115
Gender				
Male	20 (76.9%)	12 (66.7%)	8 (100.0%)	.132
Female	6 (23.1%)	6 (33.3%)	0 (0.0%)	
Race				
Chinese	16 (61.5%)	10 (55.6%)	6 (75.0%)	.508
Malay	8 (30.8%)	6 (33.3%)	2 (25.0%)	
Others	2 (7.7%)	2 (11.1%)	0 (0.0%)	
Presence of Cataract	21 (80.8%)	13 (72.2%)	8 (100.0%)	.281
Grading pterygia				
1	1 (3.4%)	1 (4.8%)	0 (0.0%)	.850
2	15 (51.7%)	10 (47.6%)	5 (62.5%)	
3	8 (27.6%)	6 (28.6%)	2 (25.0%)	
4	5 (17.2%)	4 (19.0%)	1 (12.5%)	
Preoperative Cylinder (dioptre)				
Median (Range) (*n* = 20)	−2.25 (−8.75–0.00)	−2.25 (−8.75–0.00)	−2.50 (−8.75 - −1.25)	.705
Postoperative Cylinder (dioptre)				
Median (Range) (*n* = 26)	−1.13 (−3.75–0.00)	−1.00 (−3.70–0.00)	−1.45 (−3.75–0.00)	.521
Graft Area (cm^2^)				
Total	49.50 (25.00–108.75)	49.75 (25.00-108.75)	48.00 (36.00–51.00)	.523
Temporal	25.00 (12.50–87.75)	25.00 (12.50-87.75)	25.00 (18.00–30.00)	.486
Nasal Median (Range) (*n* = 29)	22.00 (11.2–45.00)	22.25 (11.20-45.00)	20.00 (16.0–25.0)	.469
BCVA preoperatively (LogMAR)				
Median (Range) (*n* = 30)	0.18 (0.00–3.00)	0.14 (0.00–3.00)	0.29 (0.00–0.70)	.476
BCVA postoperatively (LogMAR)				
Median (Range) (*n* = 30)	0.100 (0.00–0.88)	0.05 (0.00–0.88)	0.18 (0.00–0.70)	.196
Operation time (minutes)				
Median (Range) (*n* = 30)	32.50 (22.00–80.00)	30.50 (22.00–51.00)	60.50 (43.00–80.00)	<.001

Median follow-up period is 31.5 (range 2–62) months. Attempt was made to recall patients who had less than 6 months follow-up and with nopterygium recurrence during their last follow-up period. However, one patient unfortunately passed away at postoperative month 1 and the others defaulted follow-up appointments.

A summary of clinical results obtained for both groups are also shown in Table 1. The fibrin glue group had a significantly shorter operating time compared to the suture group (*p* < .001).

For the eyes that are included, two from the suture group and one from the glue group are recurrent pterygium. Otherwise, the rest are primary pterygia.

There was only one eye in the suture group that had an increase in postoperative astigmatism, this was not seen in the fibrin glue group. Five patients in the fibrin glue group had preoperative unrecordable astigmatism, which subsequently improved significantly postoperatively.

**Figure 1.**
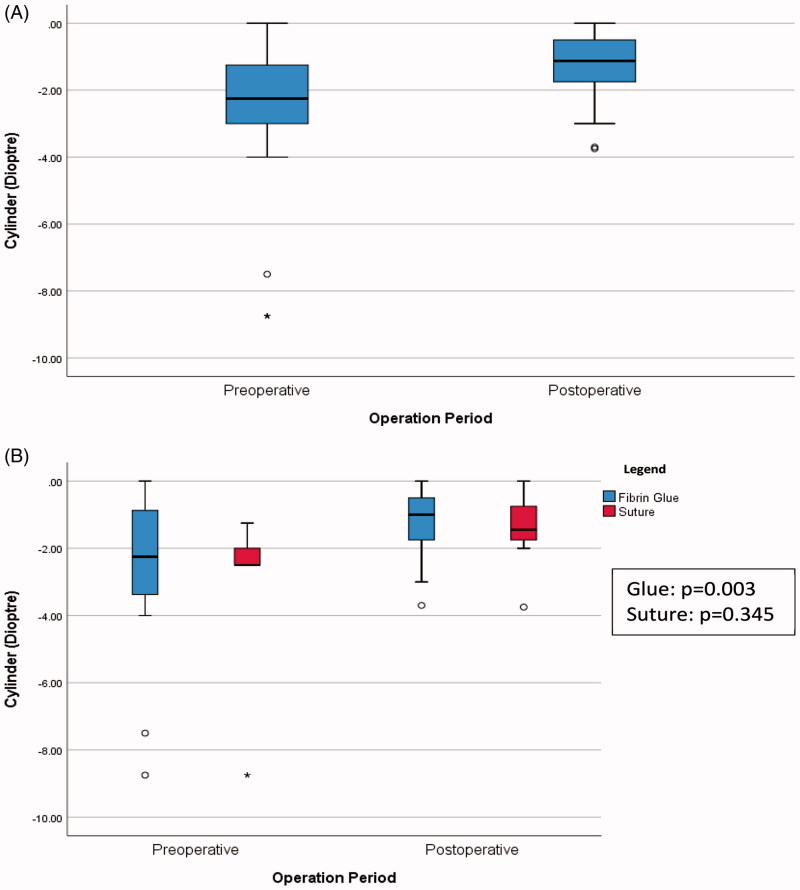
(A) Boxplot of cylinder over time (*p* = .002). (B) Boxplot of cylinder over time, comparing the two methods.

**Figure 2.**
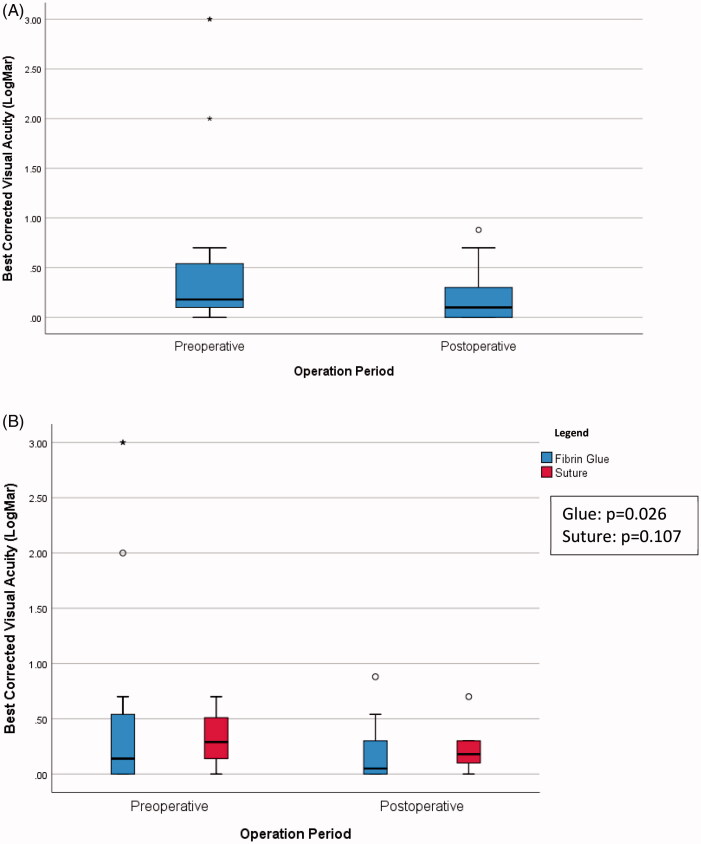
(A) Boxplot of best corrected visual acuity over time (*p* = .009). (B) Boxplot of best corrected visual acuity over time, comparing the two methods.

In Figures 1(A), 2(A), there was statistically significant improvement for overall best corrected visual acuity (*p* = .009) and cylinder (*p* = .002). Figure 1(B) showed the boxplot of cylinder over time. In Figure 2(B), there was also statistically significant improvement in visual acuity in the fibrin glue group (*p* = .026), but not in the suture group.

**Figure 3.**
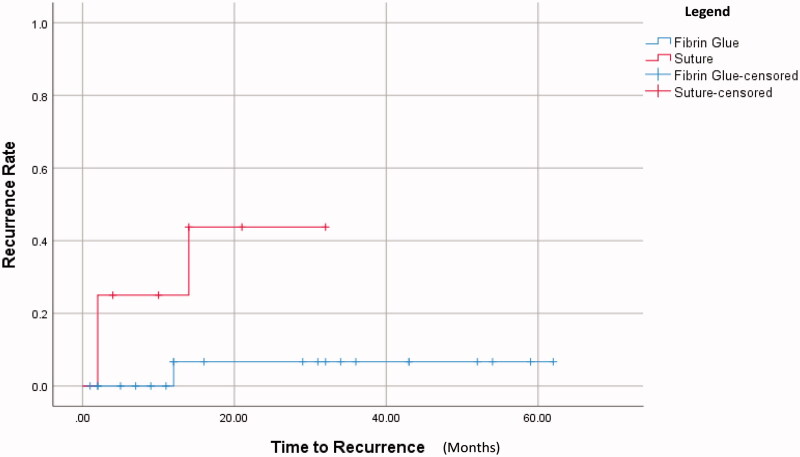
Survival analysis of recurrence rate over time. *p* = .021.

In the fibrin glue group, there was1 recurrence (4.5%) of the nasal pterygium that was noticed at the 1st year follow-up visit. In the suture group, three recurrences (37.5%) were seen of both the nasal and temporal pterygia at 2 months and 14 months postoperatively. Log-rank test showed that the patients treated by fibrin glue would have had significantly longer time duration to recurrence (*p* = .021). 1 year recurrence rate for fibrin glue group and suture group were 6.7% and 25.0% respectively, as shown in Figure 3.

**Clinical Photograph 1.**
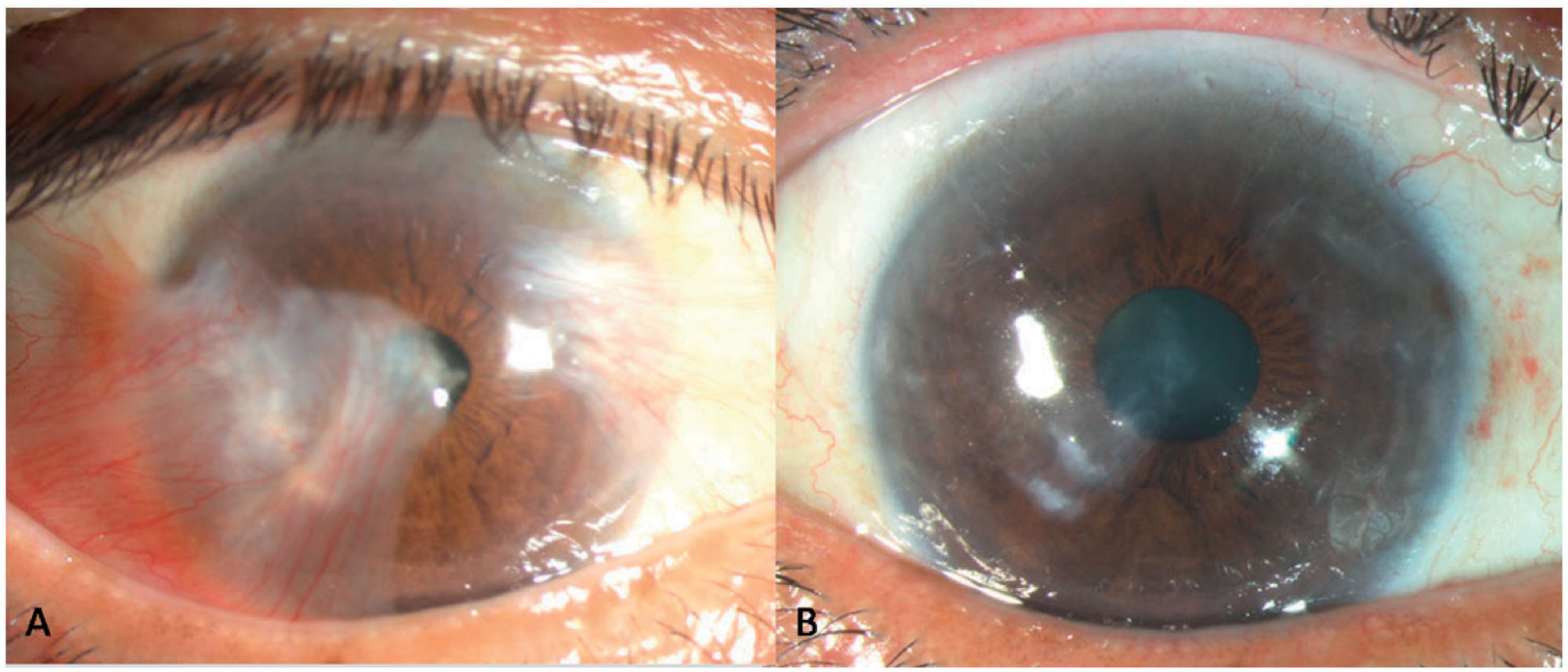
Preoperative (A) anterior segment photograph of a patient with a large double-headed pterygium obscuring the visual axis, with unrecordable astigmatism and a best-corrected visual acuity (BCVA) of 6/21. Postoperatively (B), astigmatism improved to −1D and BCVA improved to 6/6.

**Clinical Photograph 2.**
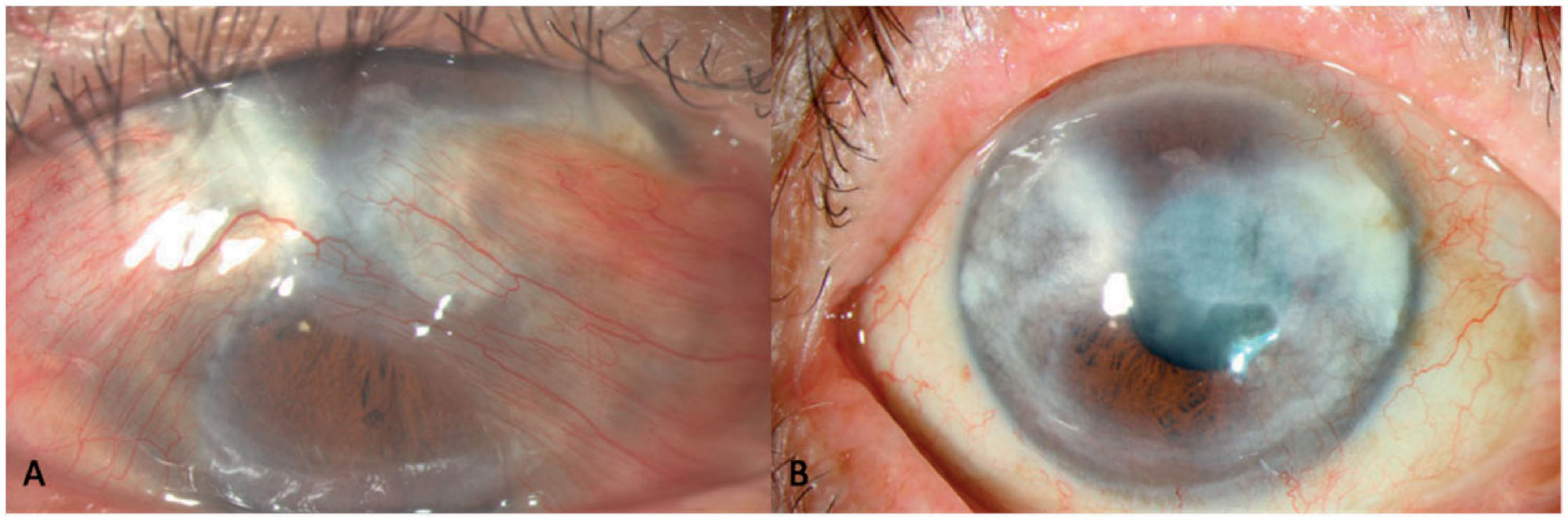
Anterior segment photo of a kissing double-headed pterygium (A) with preoperative unrecordable astigmatism and Hand movement vision. Astigmatism improved to −5.5D postoperatively (B) with a best-corrected visual acuity of 6/12 despite significant residual scarring.

None of the eyes had postoperative graft dislocation, contraction or developed limbal stem cell deficiency at last follow-up. Clinical Photographs 1 and 2 are the preoperative and postoperative clinical photographs of 2 eyes with double-headed pterygia. 

## Discussion

Using different procedures, previously published studies have shown varying degrees of recurrence that ranged from 0% to 71.42% for double-headed pterygia [8,15–17,18]. In this study, recurrence was defined as the presence of any corneal fibrovascular encroachment. There was a recurrence rate of 4.5% for the glue group versus 37.5% for the suture group, and this was statistically significant (*p* = .021). This implies that fibrin glue is superior to conjunctival suture in terms of recurrence rate. Fibrin glue has been used for many years and acts as an adhesive by activating fibrinogen with thrombin. There have been very few studies in the management of double-headed pterygium with large sample size and long-term follow up. Nevertheless, the recurrence rate of pterygium, depending on the surgery type such as glue-assisted or suture-assisted autografting, has been a matter of controversy even for single-headed pterygium. Some reports documented lower recurrence [19,20] in fibrin glue group compared to suture group, whereas other reports stated the converse [21]. Another had similar recurrence rates for both groups [22]. A study by Tarek *et al* had 0% recurrence for double-headed pterygia that underwent vertical split conjunctival autograft using fibringlue [6].

Fibrin glue can potentially reduce the amount of postoperative inflammation, which may be related to a small chance of recurrence. It has the additional benefit of reducing intraoperative time as well as improving postoperative comfort [13]. Since our surgical technique requires placement of two conjunctival autografts, increased number of sutures will be required to suture the graft in place. This can lead to an increase in postoperative inflammation resulting in increased risk of recurrence. In addition, the patients in our fibrin glue group notably had larger and more advanced pterygia with the need for larger conjunctival autografts.

Hirst et al. [23] studied the time to pterygium recurrence and found that in recurrent cases, there was a 50% chance of recurrence in the first 4 months and 97% chance of recurrence within the first 12 months. Compared to primary pterygia, recurrent pterygia had a higher rate of subsequent recurrence with a shorter interval to recurrence. Hence, our study included patients who had a minimum of 6 months follow-up. For those that recurred, a repeat pterygium removal surgery was not done as the affected patients’ best corrected visual acuity were better than 6/12.

There was statistically significant improvement between the overallpreoperative and postoperative BCVA (*p* = .009), and overall preoperative and postoperative cylinder (*p* = .002). Though a statistical improvement in BCVA was found in the fibrin glue group (*p* = .026), this was not demonstrated in the suture group (*p* = .107). One of the possible reasons, as mentioned, was that the fibrin glue group had larger and more advanced pterygia, hence improvement in BCVA was more apparent.

In this study, it was also noted that operation time was significantly shorter in the fibrin glue group. This trend was also noted in a meta-analysis done to compare the safety and clinical efficacy of fibrin glue with that of suture for conjunctival autograft attachment in single-headed pterygium surgery [12]. According to Hall et al. [22] shorter surgical time would make fibrin glue more cost-effective. It is also postulated as a contributing factor in reducing postoperative fibrosis at the donor area [6]. Our results concur with earlier reports which concluded that excision of pterygium leads to statistically significant reduction in astigmatism, which improves vision significantly [24,25].

Of note, intraoperative or postoperative complications did not occur in any of the eyes. Our suture group had a recurrence rate of 37.5%. We note that various studies have differing definition of recurrence, with some defining recurrence as presence of fibrovascular tissue crossing the corneo-scleral limbus onto the clear cornea [17]. A study by Wu et al. showed a 35% recurrence rate with the technique of conjunctival rotational autograft combined with conjunctival autograft for double-headed pterygia [16]. Adjunctive cytostatic eye drops such as Mitomycin C, 5-fluorouracil and Triethylene Thiophosphoramide have been used during surgery, to decrease chance of pterygia recurrence. These were not used in our study group. The agents are also known to lead to complications such as scleral and corneal thinning, ulceration, secondary glaucoma, palpebral hyperpigmentation and rarely even globe perforation [26–29].

Our study is limited by its retrospective nature and further by patients who were lost to follow up and had to be excluded as a result. Though larger randomized control studies are required to further address this question, it is difficult to conduct a study with adequate sample size given the uncommon occurrence of double-headed pterygia. It would be ideal to obtain data about patient’s satisfaction towards the surgery, such as the postoperative cosmetic outcomes, postoperative inflammation, pain, foreign-body sensation, etc. To avoid bias in interpreting case notes retrospectively, these parameters were not evaluated. Though our study suggests that the use of fibrin glue is superior to suture closure in terms of operative time and recurrence rate, we are limited practically by the financial constraints of the individual. In our local setting, fibrin glue is more expensive and not covered by government subsidies. As such, patients will have to spend significantly more on the surgery.

## Conclusion

In conclusion, our study has shown that lower recurrence rates and good postoperative visual outcomes can be achieved with the split conjunctival autograft technique using fibrin glue. However, larger randomised controlled trials are still required in our pursuit for the best surgical technique with minimum complications and recurrence.

## Ethics approval and consent to participate

The study was approved by National Healthcare Group Domain Specific Review Board. This board is in charge of research done in National University Health System. Requirement for informed consent was waived by National Healthcare Group Domain Specific Review Board as patient’s outcome/management was not affected and there was no direct patient contact during the project. All methods were carried out in accordance with relevant guidelines and regulations.

## Data Availability

Data available on request from the Dr Dayna Yong Wei Wei.
